# Mechanical Properties and Failure Mechanism of a Carbon Fiber/Silicone Rubber High-Temperature Flexible Textile Composite

**DOI:** 10.3390/polym18030358

**Published:** 2026-01-29

**Authors:** Jiandong Huang, Jie Mei, Hui Ning, Yue Zhuo, Hanxiang Shan, Fanfu Meng, Xueqi Jiang

**Affiliations:** 1 School of Aeronautic Science and Engineering, Beihang University, Beijing 100191, China; huangjiandong2015@126.com; 2 Science and Technology on Space Physics Laboratory, China Academy of Launch Vehicle Technology, Beijing 100076, China

**Keywords:** flexible textile composites, high temperature, mechanical properties, failure analysis

## Abstract

To optimize the aerodynamic performance of the aircraft across its entire cross-section, wing shape control must be maintained based on flight operating conditions. A high-temperature flexible textile composite, which is the key to achieving the deformation of an aircraft wing, is urgently required in the deformable structure of high-speed aircraft. In this work, a novel type of flexible textile composite with enhanced temperature resistance was fabricated by plain-woven carbon fibers coated with silicone rubber. The material testing was carried out in a wind tunnel to simulate both the harsh temperature field distribution and the mechanical loads caused by aerodynamic forces under the flight profile. For the first time, temperatures exceeding 1000 °C were attained on the windward side of an aircraft wing with a peak recorded temperature of 1600 °C. The failure mechanisms of the flexible composites are revealed, and the thermal stability of the composites is evaluated. The results show that the significant tensile anisotropy in the flexible composites is along different off-axis angles, and the failure modes also change with the off-axis angle. The material does not show significant high-temperature oxidation ablation under thermo-mechanical coupling. This work reveals that under the triple action of such high temperatures, stress caused by wing surface tensioning, and the mechanical load caused by aerodynamic forces, the failure mechanism of the flexible textile composite is dominated by the mechanical load at high temperatures rather than by thermal instability, as is conventionally claimed.

## 1. Introduction

The aerodynamic characteristics of aircraft are complex and variable. Conventional fixed wings designed with a single technical index can ensure the aerodynamic performance reaches the optimal level under specific working conditions. However, they often fail to meet the aerodynamic performance requirements across the entire cross-section of high-speed aircraft, resulting in performance degradation of the aircraft. In addition, fixed wings exhibit drawbacks such as excessive mass and difficulty in adapting to the constraints of multi-platform launches, which conflict with the need for large wingspans in high-speed aircraft and seriously restrict the overall optimal performance. Therefore, this is one of the biggest challenges for controlling an aircraft’s shape based on flight operating conditions [1].

A new type of composite material, referred to here as a flexible textile composite, is fabricated through specialized processes with high-strength and wear-resistant fibers as the reinforcement phase and a high-temperature-resistant, corrosion-resistant, and highly deformable rubber as the matrix. This material exhibits low stiffness and shows flexibility at lower stress levels, enabling wings constructed from it to be folded and unfolded, thereby facilitating aircraft storage and transport. When pre-stress is applied, the material has a specific stiffness, while under larger external loads, it exhibits high strength. The flexible wing has a leeward side and a windward side. The windward side is directly eroded by the incoming flow; therefore, it attains a higher temperature than the leeward side. Combining the above two aspects, the flexible textile composite is regarded as essential for achieving the deformation of aircraft wings and has attracted growing interest for aircraft applications [2,3].

High-temperature flexible composites serve as the key material in the deformable structure of the high-speed aircraft, such that the configuration of the aircraft can be variable. Typically, these composites are textile composites, which use high-strength and abrasion-resistant fibers as the reinforcement phase and the high-temperature resistance rubber as the matrix. In contrast to conventional engineering materials, flexible textile composites display highly complex nonlinear and anisotropic mechanical behavior. The uncertain nonlinear mechanical properties of flexible textile composites under high temperature remain one of the critical bottlenecks limiting their application in aerospace vehicles.

The flexible textile composites primarily exhibit stiffness under tension loading. Ambroziak et al. [4] conducted uniaxial tensile tests on fabrics along different directions, demonstrating the anisotropic nature of the material. Both uniaxial and biaxial tensile tests have been performed, confirming consistent tensile strength characteristics between two testing methods. Due to the soft nature of the matrix, the flexible textile composite often fails by tearing. Qu [5] used the trouser-shaped tests to characterize the failure properties of flexible composite and developed a three-stage model to analyze the failure process. They also proposed a central tearing test to examine the effect of initial cracking on the tearing [6]. A new fracture model was developed which predicts the failure strength well. Their work suggests the strong nonlinearity of the mechanical and failure behavior in flexible textile composite at room temperature. However, the high-temperature mechanical properties remain poorly characterized.

The environmental temperature significantly influences the mechanical properties of the flexible composites. As most flexible composites are not designed to be high-temperature resistant, the tests were carried out around room temperature [7,8,9]. Chenet al. [10] studied the thermal/mechanical responses and failure modes of flexible composites under oxygen methane flames, and combined with simulation, the failure mechanism of flexible composite structures under combined thermal flux and tensile loading was revealed. Qin et al. [11] carried out uniaxial tensile tests for SiC woven fabric after 1200 °C oxidation treatment. They found the transition of the deformation mechanism from fracture-before-straightening to fiber embrittlement. Similar works have been carried out for a series of composite exposed to various service environments [12,13,14]. Notably, the load was still applied at room temperature.

Ablation resistance is another important property for high-temperature applications of the flexible composites. Most ablation resistance tests on flexible composites were carried out for flammable materials with PVC being the most commonly used matrix [15,16,17]. Ding et al. [18] tested the ablation behaviors of an in situ SiC-reinforced silicone rubber and found that additional polycarbonylsilane can greatly improve both the static and dynamic ablation performance. Yang et al. [19] revealed the ablation behavior of high-silica/phenolic pyramidal lattice-reinforced silicone rubber composites. However, because the structure of textile composites is quite different from the lattice-reinforced silicone rubber composites, their ablation behavior may also be different. For textile composites, the thermal insulation performance has been extensively investigated [20,21,22,23]. Meanwhile, the ablation requires further investigation.

In this study, room-temperature tensile tests and high-temperature wind tunnel tests were conducted on a flexible textile composite. The macro/micro mechanical properties’ evolution mechanism and failure modes of flexible composites were clarified, and the long-term adaptability of high-temperature-resistant composites in different states was verified. The temperature response and surface adaptability of the flexible composites under deformation were also studied.

## 2. Uniaxial Tensile Test and Mechanical Properties Analysis of High-Temperature Flexible Composites at Room Temperature

### 2.1. Uniaxial Tensile Specimens

The test materials include three types: a carbon fiber flexible composite, a quartz fiber flexible composite, and a combination of the two. Among them, the surface of the fabric fibers is coated with W-XT silicone rubber, and a layer of polyimide aluminum-coated film is adhered to the surface of the flexible composite materials. All the tension tests in this study that were conducted referred to <Coated fabrics for membrane structures> (GB/T30161-2013) [24]. This standard stipulates that the determination of the breaking strength of membrane structures should be carried out in accordance with GB/T 3923.1 [25].

**Figure 1:**
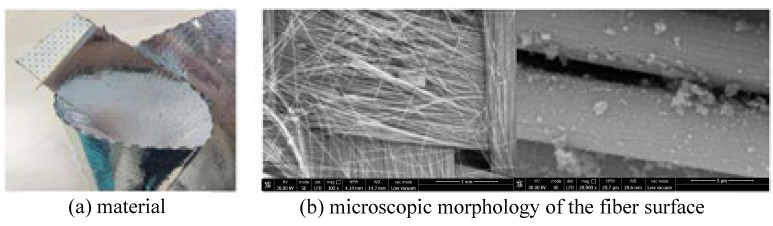
The initial material of carbon fiber flexible composite.

**Figure 2:**
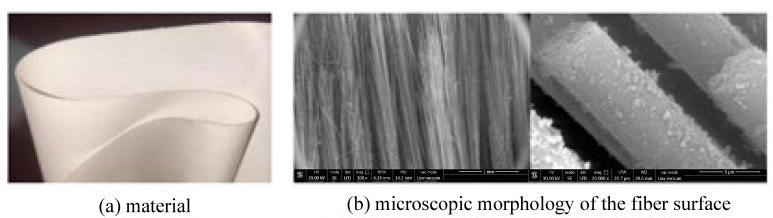
The initial material of quartz fiber flexible composite.

The main material parameters of the carbon fiber flexible composite and the quartz fiber flexible composite are shown in Table 1, and the material and the microscopic morphology of the fiber surface are shown in Figure 1 and Figure 2, respectively.

### 2.2. Uniaxial Tensile Test Results

**Figure 3:**
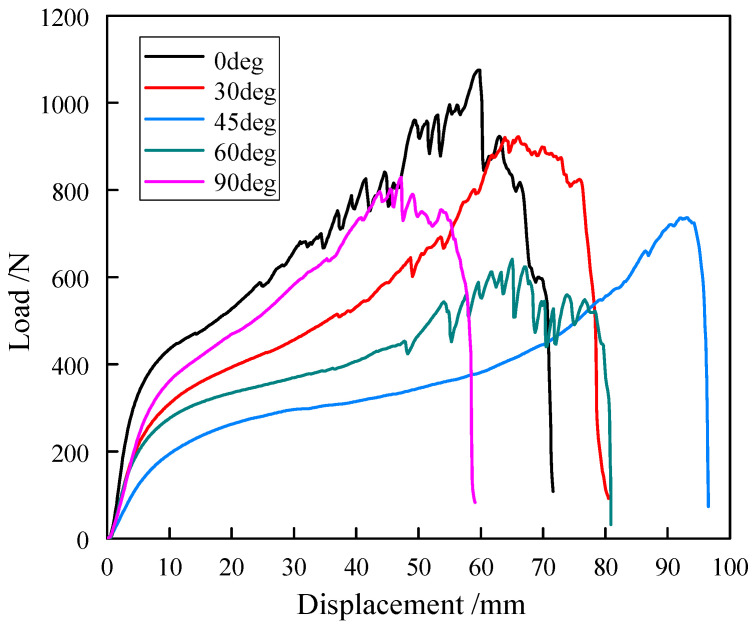
Uniaxial tensile results of the carbon fiber flexible composite specimens.

**Figure 4:**
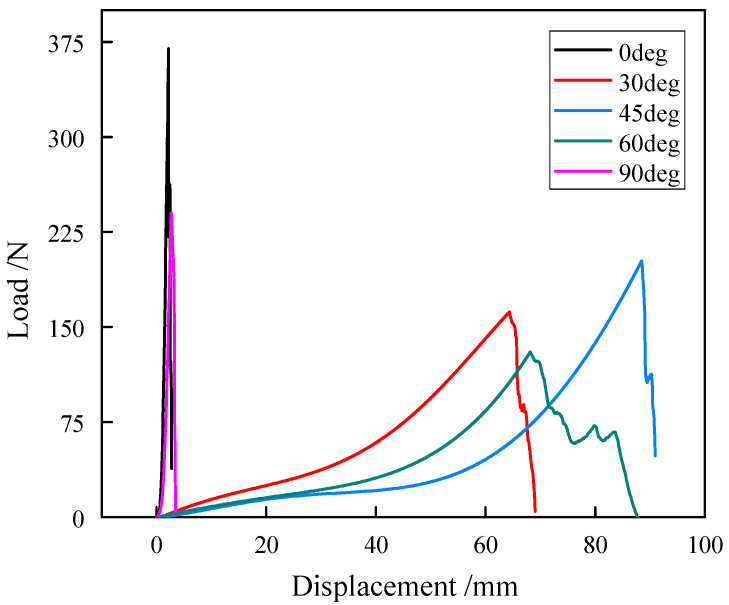
Uniaxial tensile results of the quartz fiber flexible composite specimens.

The uniaxial tensile results of five off-axis angles of the carbon fiber flexible composite and the quartz fiber flexible composite specimens are shown in Figure 3 and Figure 4. The tensile force–displacement curves of the materials clearly show different in fracture elongation across off-axis angles, indicating significant anisotropy. Moreover, the tensile force–displacement curves at various angles exhibit significant nonlinear behavior, and the degree of nonlinearity reflects the variation in the tensile stiffness of flexible composites with load.

The special weaving method causes the base fabric yarn of the flexible composites to be not straight, which is a phenomenon known as crimp. The degree of crimp of the warp and weft in flexible composites is complementary: that is, when the degree of crimp of the weft increases, the degree of crimp of the warp will decrease, and vice versa; this effect is called crimp interchange. The obvious nonlinearity of the mechanical behavior of flexible composites is caused by the interval effect between the warp and weft, the interaction between the yarn and yarn, and the interaction between the yarn and matrix.

The tensile strength of the two kinds of flexible composites is greater along the warp direction than along the weft direction. The reason is that during the weaving process of the substrate of the flexible composites, the warp yarn is generally straightened by applying pre-tension while the weft fiber is wound and braided. The initial crimp of the weft yarn is higher than that of the warp yarn, which results in the elongation at break of the flexible composite along the warp direction being smaller than that along the latitude direction. After the weft is straightened, its stretching behavior is similar to that of the warp.

### 2.3. Study on Failure Modes and Mechanisms

**Figure 5:**
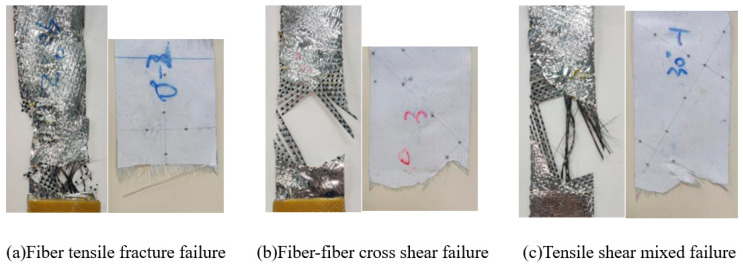
Failure modes of flexible composites.

The specimens of flexible composites show different fracture forms after being pulled apart at different off-axis angles. By observing the tensile failure process and tensile fracture of the specimens, three failure modes of flexible composites are summarized, as shown in Figure 5.

The first form of failure is fiber tensile fracture, as shown in Figure 5a, which occurs in specimens stretched at warp and zonal declination angles, representing pure tensile failure. The flexible composite is mainly subjected to normal stress. As the load gradually increases, the fabric contracts laterally. At this time, the outer polyimide aluminized film of the carbon fiber flexible composite first falls off and fractures at multiple locations, and the fiber bundles of the composite bear the main load. Subsequently, the fibers along the line between the edge fractures were broken until the specimen was completely destroyed.

The second form of failure is manifested as fiber interaction (cross shear) failure, as shown in Figure 5b, which occurs in the specimen subjected to 45-degree angle tension. The warp and weft fibers form a 45-degree angle with the direction of the tensile load, and there are few directly loaded fibers, which are uniformly symmetrically distributed. As the load gradually increases, the failure initiates at the edge fibers of the specimen with less constraint, and then it gradually falls off from the coating from both sides inward. The flexible composites undergo significant deformation, leading to the highest fracture elongation. The fracture surface of the polyimide aluminum-coated film of the carbon fiber flexible composite exhibits a basically straight fracture path.

The third form of failure is manifested as the coupling of the above two forms of failure, namely tensile shear coupling-type failure, as shown in Figure 5c, which can be observed in the specimens subjected to 30-degree angle and 60-degree angle tension. Due to the initial fiber direction of flexible composites being at a certain angle to the load direction, the main load-bearing fibers deviate, causing the angle between the warp and weft fibers to deviate from perpendicularity. The flexible composites are in a state of fiber tension cross-shear coupling. As the load gradually increases, the fibers on both sides of the specimen gradually fall off, and the middle part of the fiber breaks. The polyimide aluminum-coated film is destroyed by the detachment of carbon fibers.

## 3. High-Temperature Mechanical Properties Test and Failure Analysis of High-Temperature-Resistant Flexible Composites

### 3.1. Wind Tunnel Test Scheme

The wind tunnel test is essential for the ground-based simulation of the temperature and oxidative conditions encountered in actual flight. Its unique capability to apply one-sided heating is crucial for accurately evaluating material performance and structural response under realistic flight conditions.

The object of the wind tunnel test is the combination of quartz fiber flexible composite materials and carbon fiber flexible composites, where the outer side is quartz fiber flexible composite material and the inner side is carbon fiber flexible composite material.

**Figure 6:**
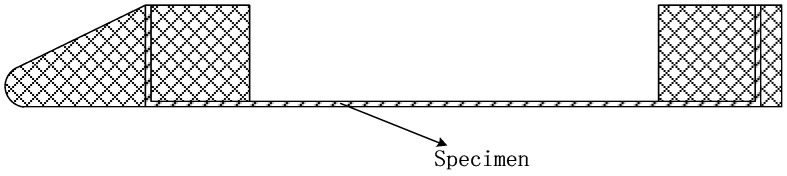
Schematic diagram of flat tooling.

The planar fixture for simulating the non-deformed state of the flexible specimen is shown in Figure 6. The specimen is connected to the fixture through bolted joints on both sides with a specified preload (50 kg) applied prior to connection. During testing, real-time monitoring of the preload is not feasible. This configuration is designed to mitigate profile deformation induced by aerodynamic loads.

**Figure 7:**
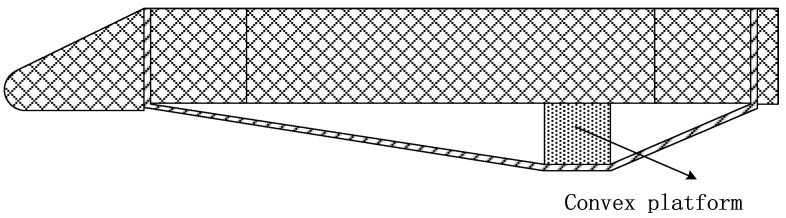
Schematic diagram of convex platform tooling.

Flexible composite materials must be supported by a rigid structure in realistic flight conditions. Under aerodynamic loading, the interaction between the two can induce coupled thermal–mechanical effects, leading to material failure. Consequently, a convex platform is designed to replicate the deformation states of flexible test specimens for accurate simulation, as shown in Figure 7. The fixture is modified from a flat test fixture by adding a metal convex platform at the middle of the flexible composite material surface. The boundary of the convex platform is processed by rounding, and the material is steel. The purpose is to simulate the deformation state of the flexible composite material and the relative relationship between the rod.

Three different states were designed for the test. Among them, the first state referred to the actual aircraft service environment conditions, and low heat flow and high heat flow were applied, respectively, in different time processes. The second state was to apply low heat flow throughout the entire process, and the third state was to apply high heat flow throughout the entire process.

### 3.2. Results and Discussion

In this section, the wind tunnel tests results and corresponding discussion are organized according to the design status. On the one hand, since the wind tunnel test of design status 2 was carried out on the convex platform, the incoming flow conditions of design status 1 and 2 were the same, but the fabric surface deformation was different, which leads to the different temperature responses. Due to the convex platform, the stress and temperature field distribution is more serious. On the other hand, the wind tunnel tests of design status 2 and 3 are both carried out on the convex platform, but the incoming flow conditions are different, producing similar deformation and dissimilar temperature responses.

#### 3.2.1. Design Status 1

**Figure 8:**
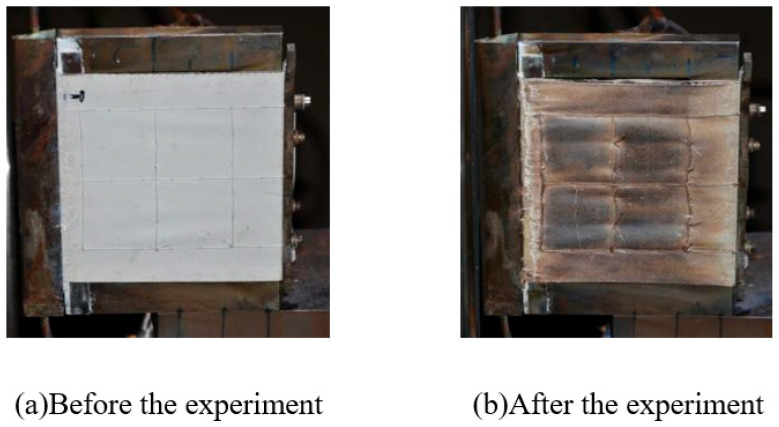
Appearance of specimen before and after Wind Tunnel experiment for design status 1.

After the specified heating period, the whole specimen remained intact, the peak temperature recorded on the windward side was about 1000 °C, and the peak temperature of the leeward side was about 600 °C. The appearance of the specimen before and after the experiment is shown in Figure 8.

**Figure 9:**
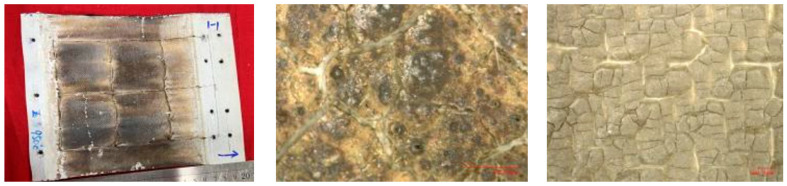
Macroscopic morphology of the specimen after wind tunnel experiment of design status 1.

The morphology after the experiment is shown in Figure 9. The overall structure of the specimen is intact with no obvious damage (cracking, fracture, etc.) observed. The windward side (except for the assembly part) is brownish black, and the leeward side is light beige. Further magnification, severe aging and cracking of the silicone rubber were revealed on both the windward and leeward surfaces with carbonization occurring on the windward side and turning black.

**Figure 10:**
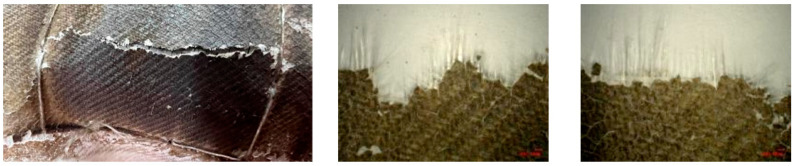
Carbon fiber flexible composite.

The heated area of the specimen was in a hard and brittle state, and the quartz fiber flexible composite on the windward side could be easily broken. When observed under a stereomicroscope, the quartz fiber fabric bundle pulled out of the fracture could be seen, as shown in Figure 10. In contrast, the inner carbon fiber flexible composite cannot be broken or torn. The cracked and aged silicone rubber on the surface can be easily removed.

Scanning electron microscopy was used to observe the microstructure of the broken fracture surface of the quartz fiber flexible composite, and the surface of the carbon fiber flexible composite was also observed. The results are as follows.

**Figure 11:**
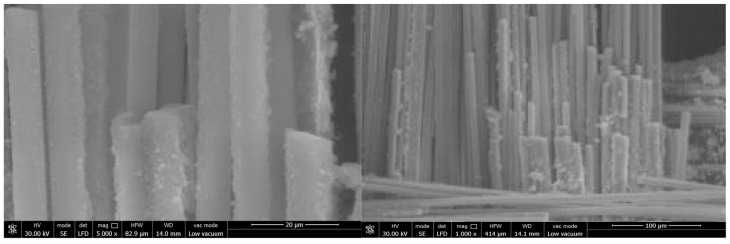
Microscopic morphology of the quartz fiber flexible composite of design status 1.

The surface and fracture of quartz fiber flexible composite are different from carbon fiber flexible composite: The quartz fiber fabric has a uniform diameter and no signs of deformation (bending, diameter change, etc.), melting, or other marks. A large amount of particulate matter adheres to the fiber surface, which should be residual organic matter after carbonization. The fiber fracture is relatively flat and mechanical with a typical morphology shown in Figure 11.

**Figure 12:**
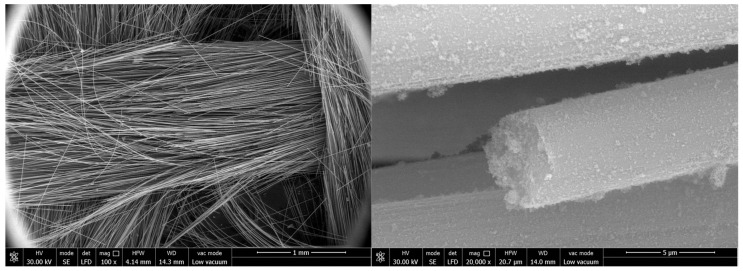
Microscopic morphology of the carbon fiber flexible composite.

Surface and fracture of carbon fiber flexible composite: The carbon fiber flexible composite fiber bundles are loose, and a large amount of organic matter residue is attached to the fiber surface after carbonization. Some carbon fiber surfaces show obvious original grooves, and no high-temperature oxidation and erosion forms such as pits or sharp shoots are observed, as shown in Figure 12.

#### 3.2.2. Design Status 2

**Figure 13:**
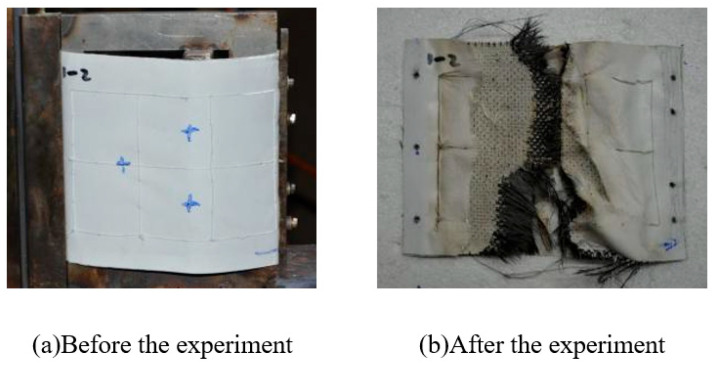
Appearance of specimen before and after wind tunnel experiment for design status 2.

After heating to a certain stage, luminous streaks emerged at the protrusion region of the specimen, which were accompanied by significant vibrational activity. Rupture failure initiated within the quartz fiber fabric layer, which was followed by subsequent fracture propagation into the carbon fiber layer. The test was terminated at 56 s due to structural failure. The peak surface temperatures reached approximately >1100 °C (windward side) and ~600 °C (leeward side) at the time of shutdown. The appearance of the specimen before and after the experiment is shown in Figure 13.

**Figure 14:**
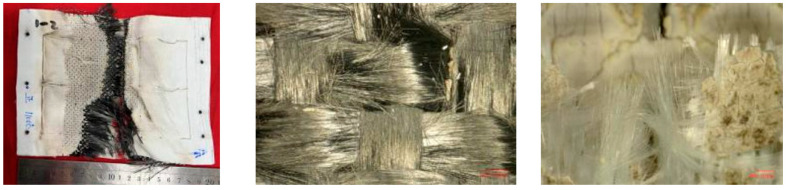
Macroscopic morphology of the specimen after wind tunnel experiment of design status 2.

The morphology after the experiment is shown in Figure 14. The specimen is cracked and damaged with severe damage on the windward side. The silicone rubber on this side is aged in beige color. The quartz fiber flexible composite and carbon fiber flexible composite on the windward side cracked at the bending point (high temperature and stress concentration), and there is a loss of about 60 mm of quartz fiber flexible composite on the windward side. The edge area of the carbon fiber flexible composite is torn, and the fiber bundles are loose and brush like. The surface silicone rubber is aging and collapsing, and there are a large number of fiber strands breaking in the middle area of the carbon fiber flexible composite that is not torn. The residual quartz fiber flexible composite fracture shows pulled out quartz fiber fabric bundles.

Scanning electron microscopy was used to observe the fracture of the quartz fiber flexible composite and the carbon fiber flexible composites as well as the microscopic morphology of the surface of unbroken carbon fiber flexible composite. The results are presented below.

**Figure 15:**
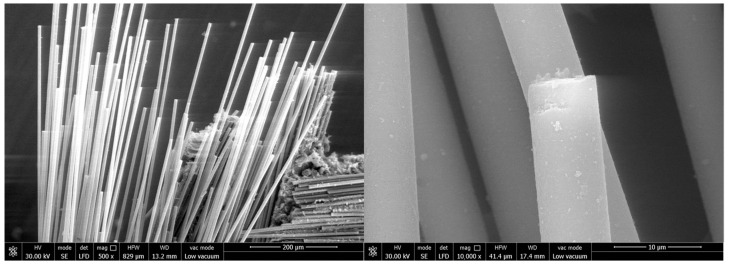
Microscopic morphology of the quartz fiber flexible composite of design status 2.

Surface and fracture of quartz fiber flexible composite: The quartz fiber fabric diameter is uniform with no deformation (bending, reducing diameter, etc.), melting or other traces. The fiber surface is attached to a large number of particles, which should be the residue of surface organic matter carbonization. The fiber fractures appeared relatively flat and mechanical with no obvious ablative characteristics. A representative morphology is provided in Figure 15.

**Figure 16:**
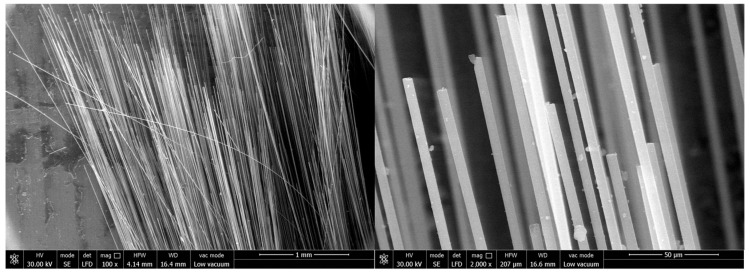
Microscopic morphology of the carbon fiber flexible composite.

Surface and fracture of carbon fiber flexible composite: The fiber bundles in the fracture area of the carbon fiber flexible composite are loose, a small amount of organic carbonization residual particles are attached to the fiber surface, and the original grooves are clearly visible on the surface of carbon fiber, without pits, bamboo shoots and other high-temperature oxidation ablation forms. In the unbroken areas, some fibers in the fiber bundle were broken, and there was no high-temperature oxidation ablation on the surface of the fibers. The fiber fractures displayed were relatively flat and mechanical, as shown in Figure 16.

#### 3.2.3. Design Status 3

**Figure 17:**
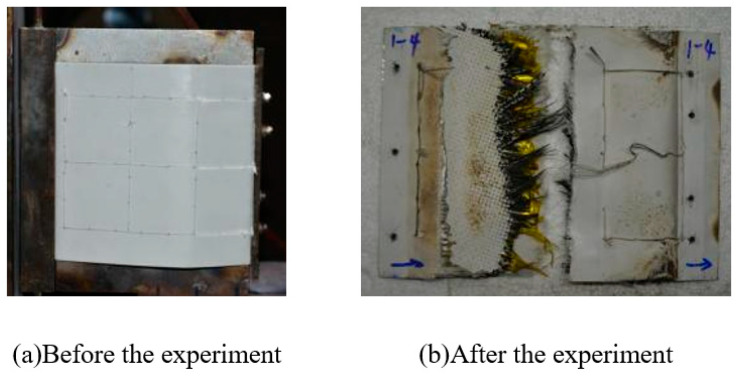
Appearance of specimen before and after wind tunnel experiment for design status 3.

After entering the flow field, severe erosion occurred at the protruding position of the specimen. The peak temperature on the windward side was about 1600 °C, and no effective data were obtained on the leeward side. The appearance of the specimen before and after the experiment is shown in Figure 17.

**Figure 18:**
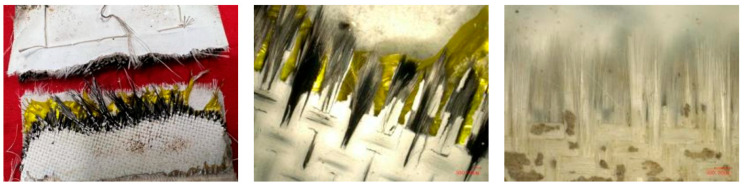
Macroscopic morphology of the specimen after wind tunnel experiment of design status 3.

The macroscopic morphology of the specimen is shown in Figure 18. The surface color of the specimen does not change significantly, and it breaks into two parts at the bending point. Among them, there is a loss of about 70 mm of the quartz fiber flexible composite on the windward side. The fiber bundles near the carbon fiber flexible composite fracture are loose and scattered in a brush-like manner, and the surface silicone rubber is aging and collapsing. The broken surface of the quartz fiber flexible composite shows pulled-out quartz fiber fabric bundles. The aging characteristics of the silicone rubber on the surface of the carbon fiber flexible composite near the fracture are not obvious, and there is aging cracking phenomenon on the residual silicone rubber on the surface of the quartz fiber flexible composite.

Scanning electron microscopy was used to observe the fracture of the quartz fiber flexible composite and the carbon fiber flexible composite, and the microscopic morphology of the surface of the unbroken carbon fiber flexible composite was observed. The results are summarized as follows.

**Figure 19:**
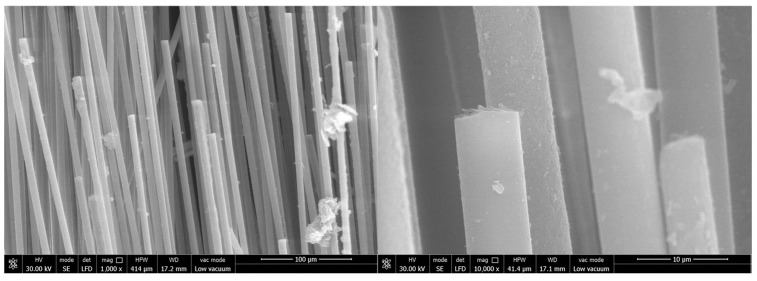
Microscopic morphology of the quartz fiber flexible composite of design status 3.

Surface and fracture of quartz fiber flexible composite: The quartz fiber fabric diameter is uniform with no deformation (bending, reducing diameter, etc.), melting or other traces. The fiber surface was attached a small amount of organic matter after carbonization particles. The fiber fracture is relatively flat and mechanical with no obvious ablative characteristics. The typical morphology is shown in Figure 19.

**Figure 20:**
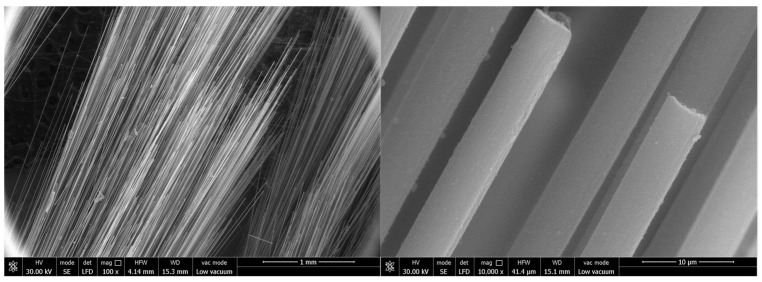
Microscopic morphology of the carbon fiber flexible composite.

Surface and fracture of carbon fiber flexible composite: The fiber bundles in the fracture area of the carbon fiber flexible composite are loose, and a small number of carbonized particles of organic matter are attached to the fiber surface. Original grooves can be clearly seen on the surface of carbon fiber, and no high-temperature oxidation and ablation forms such as pits and bamboo shoots are found. The fracture is relatively flat and mechanical, as shown in Figure 20.

## 4. Conclusions

A new type of composite material, called the flexible textile composite in this study, is fabricated through special processes with high-strength and wear-resistant fibers as the reinforcement phase and high-temperature-resistant, corrosion-resistant and highly de-formable rubber as the matrix. The material tests are carried out in the wind tunnel to simulate the harsh temperature field distribution under the flight profile. It is the first time that the windward side of aircraft wing attains above 1000 °C with a peak recorded value of 1600 °C. Uniaxial tensile tests and high-temperature mechanical properties tests were conducted on two types of flexible composites at room and high temperature. The mechanical behavior and failure modes of the flexible composites were analyzed. The conclusions are as follows:

(1) The mechanical properties of the flexible composite are related to the crimp degree of the braided structure. The nonlinear mechanical behavior of the flexible composites results from the crimp exchange effect of the yarn, the interaction between the yarn and yarn, and the interaction between the yarn and the matrix.
(2) The tensile anisotropy of flexible composites along different off-axis angles is significant, and the uniaxial tensile failure of flexible composites with different off-axis angles is different.
(3) Under the actual service environment temperature conditions without deformation, the flexible composite can be used for a long time, and no obvious damage occurred in the test part.
(4) Under the triple action of such high temperature, stress caused by wing surface tensioning, and aerodynamic loading, the failure mechanism of the flexible textile composite is dominated by mechanical loading at high temperature rather than the conventionally claimed thermal instability. In the high-temperature mechanical properties test, no obvious high-temperature oxidation or erosion characteristics were observed on the surface or cross-section of the quartz fiber flexible composite and carbon fiber flexible composite fibers of the specimens, and the fracture or cracking areas were all mechanical fractures.
(5) In the raised state, the surface of the quartz fiber flexible composite and carbon fiber flexible composite specimens form stagnation points, and the protruding position of the material is still damaged under low state heat flow condition.

## Figures and Tables

**Table 1:** Main material parameters of two composites.

Material Type	Thickness/mm	Area Density (g/m^2^)	Weave Density (yarns/cm)	
			Warp	Weft
Carbon fiber flexible composite	0.55	715	4.28	4.25
Quartz fiber flexible composite	0.28	400	20	20

## Data Availability

The raw data supporting the conclusions of this article will be made available by the authors on request.
